# Comparison between the geological features of Venus and Earth based on gravity aspects

**DOI:** 10.1038/s41598-023-39100-x

**Published:** 2023-07-28

**Authors:** Kurosh Karimi, Gunther Kletetschka, Verena Meier

**Affiliations:** 1 Faculty of Science, Institute of Hydrogeology, Engineering Geology and Applied Geophysics, Charles University, 12843 Prague, Czech Republic; 2 Geophysical Institute, University of Alaska - Fairbanks, 903 N Koyukuk Drive, Fairbanks, AK 99709 USA

**Keywords:** Exoplanets, Inner planets

## Abstract

We probe the gravitational properties of two neighboring planets, Earth and Venus. To justify a comparison between gravity models of the two planets, spherical harmonic series were considered up to a degree and order of 100. The topography and gravity aspects, including ${\Gamma }_{zz}$ (vertical derivative of the vertical component of the gravity field), strike alignment (SA), comb factor (CF), and *I*_2_ invariant derived from the Marussi tensor, were calculated for the two planets at specifically selected zones that provided sufficient resolution. From Γ_zz_ we discovered that the N-NW edge of Lakshmi Planum does not show any subduction-like features. Its Γ_zz_ signature resembles passive continental margins on Earth, like those surrounding the Indian Peninsula. Moreover, according to SA and CF, the Pacific and Philippine-North American Contact Zone on Earth indicates significantly higher level of deformation due to convergent motion of the plates, whereas the deformation level on Venus is significantly smaller and local, when considering an equatorial rifting zone (ERZ) of Venus (between Atla-Beta Regios) as diverging boundaries. The strain mode on the East African Rift system is smaller in comparison with ERZ as its Venusian analog. The topography-*I*_2_ analysis suggests a complicated nature of the topographic rise on Beta Regio. We show that specific regions in this volcanic rise are in incipient stages of upward motion, with denser mantle material approaching the surface and thinning the crust, whereas some risen districts show molten and less dense underlying crustal materials. Other elevated districts appear to be due to mantle plumes and local volcanic activities with large density of underlying material.

## Introduction

The gravity aspects^1–3^ including the gravity disturbance δg, components of the Marussi tensor or gravity gradient tensor (the second directional derivatives of the gravity potential ${\Gamma }_{ij}$ and the invariants of the Marussi tensor *I*_0_,* I*_1_,* I*_2_), strike alignment (SA), and comb factor (CF), have been employed for detection of local petroleum, metal, diamond, groundwater^4–11^, and regional exploration of the Earth’s crust^1–3,12–14^. While models from gravity measurements are gaining increasingly higher resolution, they are being explored for remote derivation of planetary material properties on a global scale not only on Earth but also on other celestial bodies. Each of the gravity aspects has its own special characteristics. The traditional δg (gravity disturbance) is often used to detect the regional isostatic conditions, elastic thickness, and density variations of the crustal materials^15^. The ${\Gamma }_{zz}$ component and *I*_2_ invariant of the Marussi tensor are two parameters, amplifying higher frequency signals. This allows their use for specification of the depth related properties of the shallower density anomalies. The components ${\Gamma }_{ij}$ allow detection of the edges of anomalous density structures and contact areas^16^. The possible structural weaknesses, deformational status of the crust and its extent, could be shown through the SA and CF parameters, under special conditions. These special properties are discussed in the paper in relation to specific geological origin. More detailed information and mathematical derivations are presented in the Supplementary File (Fig. S1).

Similarly to the analysis of the gravity field, the magnetic field can also be considered via its magnetic gradient tensor (MGT), its eigenvector properties, eigenvalues, and invariants^10,17,18^. However, the MGT application is not as practical as gravity gradient tensor (GGT), due to the complicated nature of the magnetic field. Despite this complication, the MGT tensor has a high frequency amplifying property, and could be used as a complement to the Marussi tensor in the potential field analysis.

Here we compare the geological features of Venus and Earth based on their gravity aspects. While the masses of Venus and Earth are similar, the mobility of the Venus surface and its tectonic activity are not well resolved^19^. We want to fill this gap with a comparative analysis of the gravitational anomalies of these two planets in terms of the gravity aspects. Specifically, this work focuses on the gravity aspects of purposely selected areas and examines their geological properties. The gravity aspects were analyzed based on respective gravity field datasets attained from the EIGEN 6C4 (Earth) and Shgi180ua01 (Venus) models.

For Earth, we used the combined gravity field model EIGEN-6C4^20^. This model was generated with help of satellite gravity data from the entire GOCE mission (November 2009 until October 2013). This model has a maximum spherical degree and order of 2190. EIGEN 6C4 reaches a half wavelength resolution of $5\times 5$ arcmin (approximately 9 km) on the Earth’s surface. In this study, however, we purposely degrade this high resolution in order to compare it with the lower resolution achievable for Venus (truncated to a degree and order of 100).

The gravity model of Venus, Shgi180ua01^21^, is the latest model gained from the Magellan spacecraft (1997) to an attainable maximum spherical degree and order of about 100, with a half wavelength resolution of ~ $1.8\times 1.8$ degrees (~ 190 km) over the Venus’ surface^18^. It is the maximum resolution achievable with the available data at its equator^18^. However, in some regions, the resolution decreases to degrees as low as 40^18^. It is expected that a new gravity model of a consistent higher resolution will be provided by the proposed VERITAS mission to Venus^22^.

The initial full resolutions of the Shgi180ua01 and EIGEN 6C4 gravity models are substantially different (~ 108 arcmin versus 5 arc min, respectively). Thus, considering 1.8 × 1.8 degree grid resolution of Venus (N_max_ = 100), EIGEN 6C4 for Earth is degraded to this resolution for the purpose of comparison.

### Previous geological and gravitational studies of Venus

The crust of terrestrial planets is one of the key elements for better understanding of dynamic processes on both surface and mantle. It provides a direct indication of the partial melting of the mantle which is crucial to study petrology and geodynamics of a planet.

The Magellan mission to Venus in 1989–1994 provided a global coverage of the planet and could unravel more details relative to previous missions. It revealed that radially symmetrical geological features (coronae) are dispersed over the planet in linear zones. Whether these are locations of mantle upwelling or downwelling was unclear. A detection of long rift zones (thousands of kilometers) connected highlands with their major volcanoes. However, there were no observations of convergent plate boundaries and oceanic spreading on Venus, a planet approximately the same size as the Earth^23^. Shield volcanoes and vast volcanic plains are significant geological features on a global scale, and the composition of Venus’ mantle is assumed to be peridotite^24^. Igneous rocks on the Venus’ surface are primarily basaltic^24^. Shield volcanoes show shallow slopes, comparable to terrestrial basaltic shields, while Venus’ lava plains are analog to extensive flows of basaltic fluid lava. Only some small volcanic constructs could possibly be composed of more silicic lavas^25^.

The Magellan mission supported previous findings about the paucity of the impact craters. Their number has been estimated at ~ 1000 (this figure compares with ~ 200 terrestrial impact structures). Such a scarcity leads to the estimation of a relatively young average age of ~ 750 m.y. for the crust on Venus compared to much older surface features on Mars and our Moon^26^. In addition, the homogeneous distribution of the density of impact craters and similar crater degradation characteristics means that geological units are of similar age. Therefore, it was suggested that the surface must have been catastrophically produced in the last hundreds of millions of years, with slight changes since then^19^. It was further found that the craters are randomly distributed across the Venus’ surface but are not randomly distributed with respect to geology. Regions of volcanic shields, flow fields, coronae, and rift systems show a lower crater density, which indicates recent or ongoing resurfacing in those areas^27^.

The Venus’ convective cells have been suggested to be around 600–900 km wide^28^. Its lithospheric and crustal thickness have been estimated differently; from 100 to 500 km for lithosphere, and from 8 to 60 km for the crust^29,30^.

The elevated zones on Venus are deemed to be due to either tectonic thickening of the crust above mantle downwelling^31,32^, or due to upwelled mantle plumes^33^. One of the elevated zones is western Ishtar Terra, which covers an elliptical area of 1800–2700 km and includes the 3.5–4.5 km high Lakshmi Planum plateau. The plateau is surrounded by the highest folded mountain ranges on Venus with elevations/altitudes of 6–12 km. Due to the presence of mountain belts, its general topographic shape, and the absence of major rift systems, Lakshmi differs from other plateau-like highlands on Venus^34^. Further, with its dimensions and geomorphology, Lakshmi resembles the Tibetan Plateau and Himalaya Mountain complex on Earth^35,36^. The radar image and Bouguer anomaly of Magellan^37,38^ indicate thrust faulting, folding, and transcurrent and transpressional shear zones comprising Lakshmi Planum which acts like a rigid craton-like body thrusting into its surroundings.

The massive volcanic highlands on Venus include Beta Regio with a complex geologic history and several volcanic and tectonic stages. It is an elliptically rock-dominated structure^39^. Beta Regio probably formed due to uplifting and shows mantle heterogeneities^40^. The altitude in Beta Regio exceeds 5 km at Theia Mons and 4.7 km on Rhea Mons^39^. The area is cut by the deep Devana Chasma rift valley, formed during a phase of extensional tectonics, and coincides with the N-S axis of the Beta rise^41^.

Rift zones in general are widespread over the surface of Venus and are predominantly situated in the equatorial region. They are often associated with large volcanos and lava plains^42^. They were found to intersect all structural and material units except for the youngest lava plains. Two structural facies can be distinguished: rift valleys and graben belts. A close association between dome-shaped rises and rift valleys can be observed. They extend from the top of such rises and are generally associated with volcanic edifices. On the contrary, graben belts, which are more widespread on Venus, occur far from rises and volcanic edifices are absent in their spatial association^43^. However, both types represent zones of extensional tectonics which formed in the late times of the planet’s geological history^44^. Cracking of the lithosphere above upwelling mantle diapirs is considered as the likeliest rift-forming mechanism^45^.

Another key question concerns the mobility of the Venus’ surface and the planet’s tectonic activity. Several studies have shown that currently there is no clear evidence of modern plate tectonic activity like on Earth. Harris & Bédard^37,38^ probed the mechanisms generating shear zones with a stagnant lid mode due to mantle plumes. A recent new scenario is a horizontal traction on the root of the continents^41–44^. It was found that the active mantle convection upwelling is required for observations made on gravity and topography beneath large volcanic provinces such as the Atla Regio and Beta Regio, as well as the Devana Chasma rift^46,47^.

While a recent surface motion on Venus is limited, a large degree of lithospheric mobility in the earlier history of Venus is preserved in the planet’s geological record. Some indications are the extensive crustal thickening that forms Lakshmi Planum, which implies 2000–3000 km of crustal convergence or even more to explain the mountain belts, as well as the lateral transport and assembly of three distinct tesserae blocks to form the Tellus Regio^48^. Byrne et al.^49^ reported on a recent finding about a globally distributed set of crustal blocks in the lowlands of Venus, showing evidence of rotation and lateral movement relative to each other. They proved that lithospheric stresses are sufficient to drive a brittle failure in the upper crust. This confirms that the interior convective motion is capable of driving deformation at the Venus’ surface^49^. Those results provide a concept of the Earth´s mobile-lid tectonics (modern plate tectonics) at the one end, the static stagnant-lid regime like on Mars and Moon at the other end, with being Venus in between. According to Weller and Kiefer^35^ it is even possible that Venus is still in the transition phase from a mobile- to a stagnant-lid regime. This could be an indication that the mobile crustal blocks on Venus are the last remnants of a more global system of mobile plates on Venus.

Using the maximum eigenvalue of the horizontal gravity gradient, Andrews-Hanna et al.^50^ examined the gravity responses of equatorial Chasmata, Artemis Corona, and Ovda Regio.

We focus on the ${\Gamma }_{zz}$ signal from both the Eurasian-Indian Contact Zone (EICZ) and the Lakshmi Planum-Akna Montes-Freyja Montes Contact Zone (LAFCZ) in this study. We employ SA and CF to examine the deformational extent over the planets’ crust and analyze the potential magma plumes under Beta Regio using the *I*_*2*_ parameter. The mathematical derivations are included in the Supplementary File.

## Results

**Figure 1:**
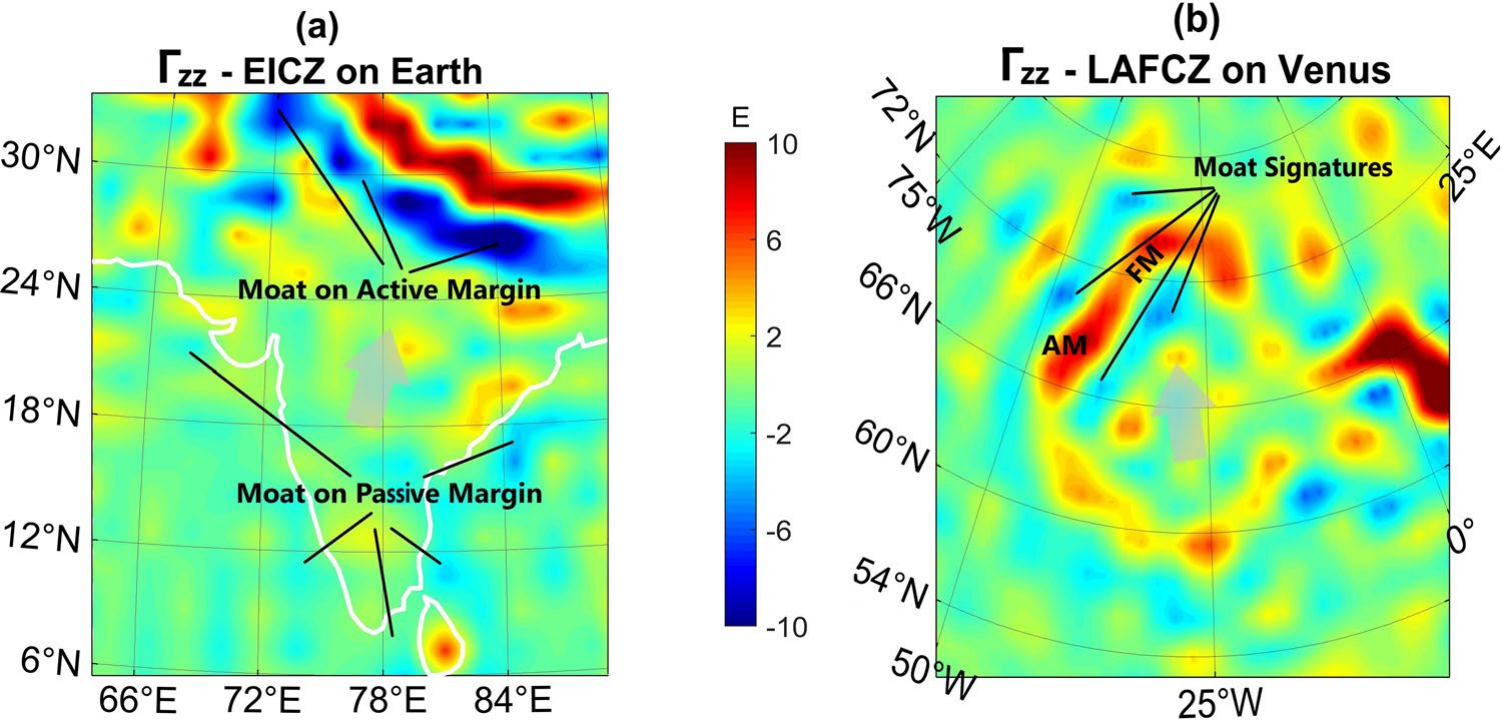
Γ_zz_ parameter of (**a**) south of Eurasia and Indian Contact Zone (EICZ). Both positive and negative Γ_zz_ show strong signals and represent the overriding and underthrusting plates in the contact zone, respectively. In contrast, the passive margin of the Indian plate has low signals. (**b**) Lakshmi Planum-Akna Montes-Freyja Montes Contact Zone (LAFCZ) within Ishtar Terra in the northern region of Venus. Γ_zz_ is weak for Akna Montes (AM) and Freyja Montes (FM) and their neighboring moat zone. The gray arrows show how the craton indents into its surroundings. The Γ_zz_ level of the moats around the southern margin of the Indian craton is comparable with the moat on the northern and north-western edge of the Lakshmi Planum.

The Γ_zz_ parameter of EICZ is shown in Fig. 1a with outstanding positive and negative NW–SE strips related to the uplifted northern and underlying southern plates, respectively (reaching more than $\pm 15 \mathrm{E}$ at some locations). Γ_zz_ in the passive continental margins surrounding the Indian Peninsula barely reaches − 5E. Figure 1b indicates Γ_zz_ of LAFCZ in Ishtar Terra on Venus. The positive narrow signal surrounding Lakshmi Planum is related to the mountain range. There is a slight negative Γ_zz_ signal surrounding the moat of the Akna Montes (AM) and Freyja Montes (FM) around Lakshmi Planum. The Γ_zz_ parameter in S and NW moats of FM, and E and W moats of AM is below − 4 E. Allowing for the dimensions (elevation > 5 km) of these mountain ranges, their average Γ_zz_ signal is weak (~ 5 E) in comparison with the Himalayan area with an average elevation of more than 5 km and average Γ_zz_ about 10 E.

**Figure 2:**
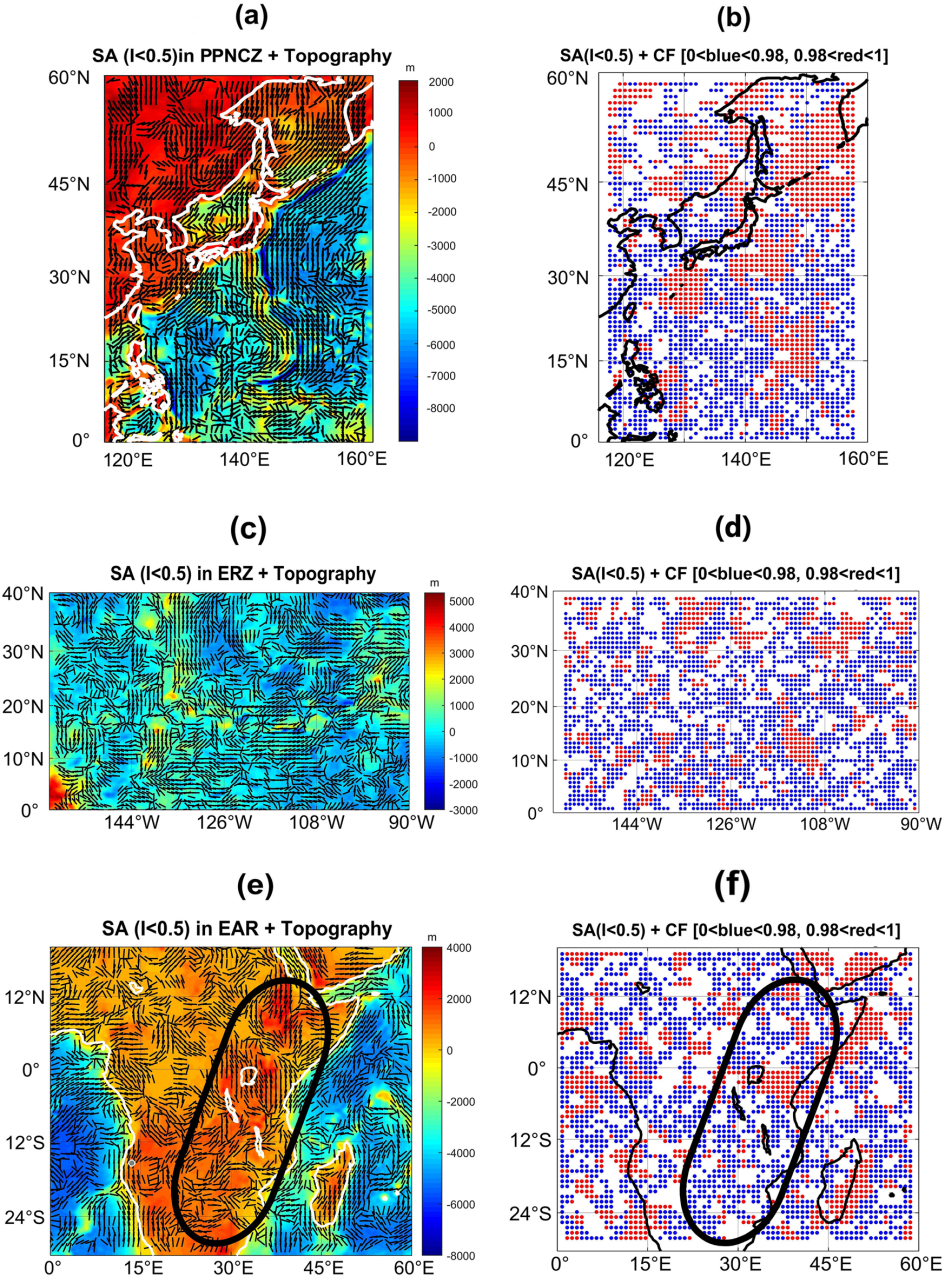
Selected zones with strike Alignments (SA) plotted on topography for (**a**) Earth in the Pacific plates and Philippine-North American Contact Zone (PPNCZ); (**c**) Venus in the equatorial rifting zone (ERZ) between Atla-Beta (AB) Regios; (**e**) Earth in East African Rift (EAR). The comb factor (CF) for (**b**) Earth in PPNCZ; (**d**) Venus in ERZ; and (**f**) Earth in EAR. CF is indicative of the strained regions affected by the convergent and divergent stresses. The ovals in (**e**) and (**f**) show the East African Rift area. The margins of the continents on Earth are ploted in black or white.

The SA solution, fitted on the topography map on three sutured regions of the planets, is plotted in Fig. 2a,c,e for I < 0.5 (I < 0.5 indicates semi-2-dimensional structures^5^). These three zones are Pacific plates and Philippine-North American Contact Zone (PPNCZ) on Earth, the Equatorial Rifting Zone (ERZ)—Hecates, Devana, Fea F, and NW part of Parga Chasmata—between Atla-Beta (AB) Regios on Venus, and the East African Rift (EAR) on Earth. The reason for selection of I < 0.5 is that values greater than 0.5 may not depict the true strike alignment of 2-dimensional structures (rift/trench/moat/fold) and their extent (for more information see the Supplementary File). To sketch the SA at each data point, we drew a small black line pointing to a direction along which the gravity parameter is most stable. This stability indicates the strike of the structures built due to the stresses exerted on the planets. The three areas of the planets are also analyzed in terms of their CF (Fig. 2b,d,f). CF shows the degree of alignment of the SA solutions in and around the planes of faults, folds, and moats. CF lies between 0 and 1. CF = 0 applies for the condition with the most non-aligned SA solutions while CF = 1 for those perfectly aligned. To see how CF is calculated, refer to the Supplementary File, and also Kletetschka et al.^51^. The strike directions in the PPNCZ are parallel to a great extent. This alignment coincides with the trenches and neighboring uplifted plates over the volcanic islands and outer rise on both sides. On the planetary scale, SA usually indicates the structural weakness provided that Γ_zz_ < 0. Of course, the geology of the area under investigation is crucially important in the interpretation of this parameter. SA with Γ_zz_ > 0 may point to the structural weakness under special conditions, e.g., when intrusive igneous materials fill the faults and fractures of sedimentary layers. The combed zones of the Earth’s trenches in the PPNCZ appear to be wide and long (red dots in Fig. 2b). Red dots indicate high CF values, > 0.98, whereas the blue dots indicate CF < 0.98 (Fig. 2b,d,f). Regarding ERZ on Venus, the parallel SA solutions (CF > 0.98) are limited to small regions. The CF spread is sparser and more limited than the trenching zone on Earth (Fig. 2c,d). Aside from the rifting zone, the parallel strike directions and high CF in the right part of Fig. 2c,d (longitude = [105° W, 90° W], latitude = [15° N, 40° N]) correspond to the interior valleys and moats around the western margin of Beta Regio. EAR is shown in terms of SA and CF in Fig. 2e,f, respectively. The ovals drawn in these Figures encircle the rifting system. Comparison of the high CF values (red dots) in Fig. 2d,f over ERZ and EAR reveals that the rifting system on Venus is wider and sparser.

**Figure 3:**
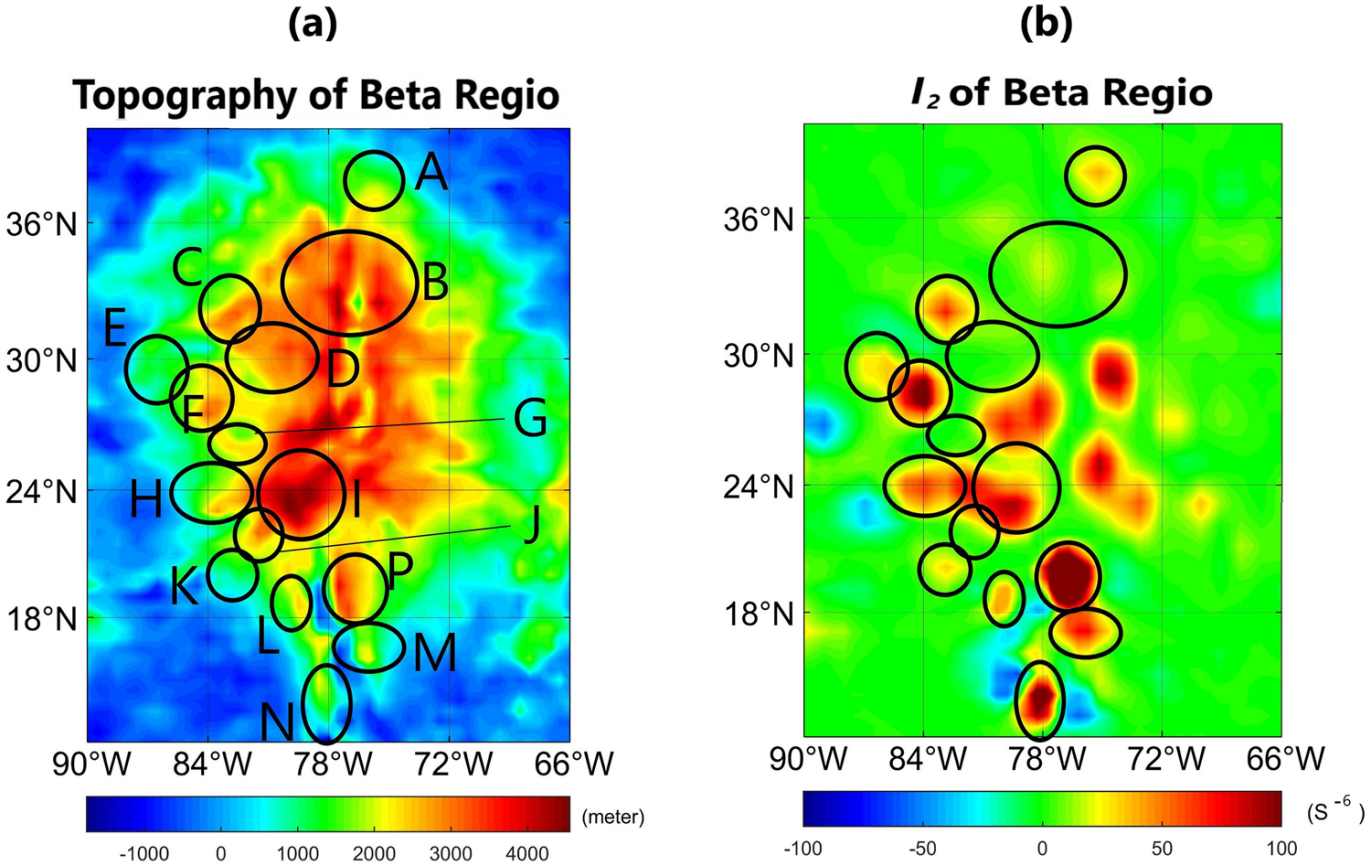
(**a**) topography; (**b**) *I*_2_ maps of Beta Regio. The topography and *I*_2_ parameters are not consistent in many areas. Detailed information is in the text.

We utilized *I*_2_ parameter to amplify the highest frequency/shallowest signals and infered the density distributions and variations in Beta Regio on Venus (Fig. 3). We divided Beta Regio into certain sectors, encircled and labelled as A, B, C, D, E, F, G, H, I, J, K, L, M, N, and P (Fig. 3). These areas are categorized into 3 groups. In group 1, including B, D, G, northern I, and central J, the *I*_2_ parameter is small (less than 30 S^−6^) whereas the elevation is high. In group 2 (like A, E, western H, K, western L, M, eastern P and N), the opposite condition holds true, i.e., the *I*_2_ signal is large despite the rather small positive elevation. Group 3 comprises areas where the topography and *I*_2_ correlate well: eastern P, M, central C, western F, eastern H, central I, and the other central and eastern areas of the map with a large *I*_2_ parameter that are not encircled.

## Discussion

The Γ_zz_ map of Earth (Fig. 1a) in EICZ reveals elongated areas of maxima and minima that coincide with plate boundaries, the areas with active orogenesis. This zone is a variation of the subduction process, whereby the subduction zone is destroyed, resulting in an orogenesis where two continental plates are sutured together. Along these stretched patterns, Γ_zz_ indicates subduction of one plate beneath the other, with Γ_zz_ < 0 and Γ_zz_ > 0, respectively. The overriding crust (Himalayas) in this condition has been folded and elevated (Γ_zz_ > 0)^52^. Generally, the main difference between Γ_zz_ signature of the active and passive boundaries on Earth is that the positive–negative strips over the active boundaries are about an order of magnitude larger than over the passive ones (Fig. 1a). Another discrepancy is that, in case of destructive borders, island arcs and related volcanism emerge on the overriding plate, increasing the level of gravity and Γ_zz_ thereof. These parameters in the boundaries are also intensely influenced by the existing vertical stresses between the contacting plates, which cause the margins to interact, thereby aggravating the isostatic disequilibrium. This is the case at EICZ, where the Γ_zz_ parameter rises (~ + 15 E) and drops (~ − 15 E) at the northern and southern edges, respectively (Fig. 1a). Hence, the intensity of Γ_zz_ can be considered for discrimination between passive and active plate boundaries.

Because of the excessive mass deficiency over the moat basin in EICZ, the gravity is negative. This effect is related to the downward stress caused by the overriding plate which pushes the dense mantle material away. Consequently Γ_zz_, which is sensitive to these shallow mass variations, has a strong negative signal in this region. As to the depressed zones of the passive margins, this mass deficit is smaller because these troughs are shallower and subsequently filled with low density sedimentary deposits. Moreover, the lithospheric stress is much smaller in these regions and thus the passive margins around the Indian Peninsula are characterized by weaker negative Γ_zz_ (from − 2 to − 5 E) (Fig. 1a).

The negative Γ_zz_ anomalous zones on Venus are mainly associated with rift valleys and moats around the mountains. Previous studies on the formation of Lakshmi Planum resulted in two models. The first, divergent model considers Lakshmi to result from mantle upwelling or rather the rising and subsequent collapse of a mantle diapir^53^. The other model explains its formation by mantle downwelling, convergence, under-thrusting and possible subduction, whereby the mountain ranges, the high topography, and the volcanic centers in the middle are considered as key features^54,55^. From the analysis of Γ_zz_ in LAFCZ and EICZ (Fig. 1), both Lakshmi planum and the Indian Peninsula show similar features. They exhibit low values and smooth variations of Γ_zz_, indicating much smoother topographic/mass variations with respect to their margins (Fig. 1). This supports the fact that these two craton-like slabs are probably older than the folded and faulted suture zones embracing them^40^. Nonetheless, there is a fundamental difference. The negative value of Γ_zz_ in the northern and western moats of Lakshmi Planum barely reaches − 5 E, which is comparable in magnitude with Γ_zz_ values of passive plate boundaries on Earth like those surrounding Indian craton, not the EICZ (Fig. 1). Such a weak signal could be related to mild flexure of the crust creating moats on the foothills of FM and AM.

Furthermore, the poorer Γ_zz_ response of FM and AM with respect to Himalayas, despite having more or less the same dimensions, may point to the hypothesis that FM and AM lack the agents that cause larger value of Γ_zz_ over these highlands. These gravity raising factors on Earth are the vertically upward stress from the under-thrusting plate, higher density of the subducting lithosphere with reference to its surrounding mantle, and petrological changes leading to larger density in the subduction zone^56^. Γ_zz_ analyses show no clear evidence for subduction at Lakshmi Planum; the presence of an ancient craton-like tessera massif in the core of Lakshmi is also not consistent with mantle diapir models. Additionally, the absence of a rift system, which is the natural consequence of surface growth due to diapiric rise, the apparent migration of volcanic activities towards its center and the abrupt termination of mountain range ridges at the edge of Lakshmi are contradictory to divergent model predictions, whereas convergence models are consistent with geological and tectonic observations^34^. Those findings strengthen the assumption of a more active tectonic regime with elements of modern plate tectonics in the very beginning of Venus’ geological history when Lakshmi Planum may have formed. Nonetheless, our calculated Γ_zz_ parameters indicate that the indentation of Lakshmi Planum is probably not accompanied with a subduction on the scale of subductions on Earth, and that mechanisms of plate tectonics were significantly restricted, probably not forming a global system like on Earth.

Although we considered Γ_zz_ for some other areas on Venus like the Artemis Corona in the southern part of Ovda Regio, it has not been discussed because of the very low resolution of the gravity model (N < 60) in this area and disruption along the observed satellite track lines.

SA and CF solutions show strain regime variations for two discrepant types of tectonics (convergent plates on Earth and divergent plates on Earth and Venus). The EAR system, the largest rifting system on Earth, is much smaller than those on Venus, and its SA and CF do not reveal a stress–strain regime comparable to those on Venus, on the scale of this study (Fig. 2c–f). The SA solution for the PPNCZ on Earth coincides with the trench plane and its neighboring ocean-ward and land-ward linear elevated areas on either side (Fig. 2a). The wide and large combed area (CF > 0.98) in the proximity of subduction zones in PPNCZ implies that the governing stresses by virtue of converging motions of the plates influence the crust to a very large scale. The resulting strain extends up to hundreds of kilometers from the trench over the volcanic highlands, as well as to the outer rise on the ocean-ward side of the trench. Hence, the SA and CF solutions are used as tools to demonstrate the crustal susceptibility and vulnerability against the exerted stresses (Fig. 2a,b). Oppositely, the combed zones on Venus within ERZ (with an opposing tectonic regime) and its proximity are smaller and more variable. This is a characteristic feature of divergent boundaries, where the stresses are much smaller than those on the convergent boundaries. Figure 2c shows that SA in ERZ on Venus are shorter and narrower. The zigzag pattern of the SA solution in this area indicates that, unlike the subduction zone on Earth, the stress direction and its spread is more local and variable (Fig. 2c,d). SA and CF are even more insignificant for EAR on Earth, implying that the deformational process is weaker throughout the diverging boundaries on Earth than on Venus (Fig. 2e,f). Generally, the high CF areas on Earth are in linear and curvilinear arrangements along folded and faulted mountain chains and plate boundaries, while on Venus, large values of CF (> 0.98) surround volcanic constructs in the moats around them in a circular pattern and along the rifted zones, where the crust is thermally uplifted^41^.

Topography and *I*_2_ parameters for Beta Regio are plotted in Fig. 3a,b, respectively. From *I*_2_ it is possible to detect the shallowest and/or densest anomalies. As *I*_2_ is extracted from the free air gravity parameter, it is highly influenced by topography. Therefore, it is expected to see the largest topographic features in *I*_2_ maps, unless the underlying mass is so small that it counteracts the gravity signal due to the topography. Because of the *I*_2_ nature, the highlands and lowlands should indicate the strongest positive and negative signals, respectively. If the dimensions of individual topographic features are comparable, only under the condition of having different densities, *I*_2_ signatures are different. Thus, for topographic districts with similar dimensions, the higher the density, the stronger the *I*_2_ signal (vice versa in case of low-density areas).

The nature of topography and the complex interrelation of volcanism and tectonism of Beta Regio implies that it was not formed by simple updoming but by a combination of dynamic support of topography together with variations in crustal thickness. The mantle plume model was found to account best for the observed geology in this area^41,57^. Based on the theory of active mantle plumes beneath Beta Regio, we interpreted our results as follows. Group 1 areas show two different phenomena: a rise in topography and partial melting of the lithospheric crust. As the lithosphere is in a molten and hot stage, its density is reduced compared to cold solid lithosphere. This causes the observed pattern of low *I*_2_ signals, i.e., low density, despite the elevated topography. Group 2 areas represent the first stage in the updoming process, in which the mantle starts to rise and gets closer to the surface, thereby causing crustal thinning and density anomalies due to higher densities in mantle material compared to densities of the lithospheric crust; despite that the actual updoming of the area, i.e., a topographic rise, is not yet visible. This relates to the observed low topography but high *I*_2_ signals in respective areas. Areas belonging to group 3 represent zones in which a topographic rise due to ascending high-density mantle plumes is already noticeable. Therefore, both topography and the *I*_2_ signal are high.

Our assumption also matches the regional distribution of groups 1 to 3 for Beta Regio. The area I comprises the Theia Mons volcano. Its SW part belongs to group 3 and shows a significant rise in altitude due to the underlying mantle plume. In the N part, however, the *I*_2_ signal is low which indicates that hot magma of lower density is present (group 1). The area B (also belonging to group 1) is cut by the Devana Chasma Rift. It was found in previous studies^41,58^ that the rift is the result of a hotspot in the northern segment (well visible in the area B) and another hotspot at its southern end in Phoebe Regio. Additionally, Kiefer and Swafford^58^ reported that hot low-density material occurs in the mantle beneath Devana Chasma and the crust is thinned relative to its surrounding areas. This is also visible in our maps in respective areas and consistent with our theory. The area P shows a prominent example of group 3, indicating topographic rise due to the underlying high density mantle material. The area N, on the contrary, shows the presence of a dense mantle plume which has not yet caused a significant rise in topography. Other areas such as F, H, and K show minor effects of the above stated phenomena in peripheral regions of the mantle plume and volcano.

Geophysical three-dimensional modeling of the lithosphere suggests that the uplift is still active at present day^59^, which indicates that plume activity and dome-formation process are still ongoing. The regional plains in the north and the area west of the rise are considered to be unaffected by the plume^41^. This is also consistent with the findings of our study (see Fig. 3).

## Concluding remarks

We applied four parameters -${\Gamma }_{zz},$ strike alignment (SA), comb factor (CF), and *I*_*2*_, derived from Marussi tensor, to interpret and compare geology of selected zones on Earth and Venus. To make this comparison reasonable, we degraded the resolution of the gravity model of Earth to that of Venus with spherical harmonic degree and order of 100.

The boundaries on the Indian-Eurasian Contact Zone (EICZ) and Lakshmi Planum-Akna Montes-Freyja Montes Contact Zone (LAFCZ) show elongated Γ_zz_ patterns. In these cases, isostatic disequilibrium and petrological changes could be the cause of an elevated Γ_zz_ level in EICZ. On passive margins of the Indian Peninsula, due to lack of these factors, the Γ_zz_ level is low. Comparison between Γ_zz_ of LAFCZ and EICZ indicates that although Lakshmi has been proposed to indent into its northern district, it does not show any overriding-underthrusting behavior, but rather its Γ_zz_ signature resembles that of passive margins around the Indian Peninsula.

SA and CF of destructive margins for Earth and constructive boundaries for Earth and Venus were analyzed to show the level of deformation in the areas inspected. According to these parameters, the Pacific and Philippine-North American contact zone on Earth indicates robust deformation due to a convergent motion of the plates, whereas the strain is quite small and local in the equatorial rifting zone on Venus when considered as constructive margins. The strain–stress regime is even weaker for the East African Rift zone on Earth. This suggests that the deformational process for divergent boundaries on Earth is weaker than on Venus.

The topography-*I*_*2*_ analysis shows the complicated nature of topographic rise on Beta Regio. According to our study, some regions are in incipient stages of upwelling, where the denser mantle material approaches the surface and thins out the crust, while some uplifted districts have their underlying crustal materials molten and less dense. Finally, some elevated districts with higher density may have been caused by mantle plumes and volcanic activities.

## Supplementary Information

Supplementary Information.

## Data Availability

The datasets generated and/or analysed during the present study are available in the [Planetary Data System (PDS)] repository, [https://pds-geosciences.wustl.edu/]. Matlab-based Graflab software was used for calculation of the Marussi tensor derivatives^60^. I_2_, SA and CF were calculated using Matlab programming software.
