# Supplementary material for: Comparison between the geological features of Venus and Earth based on gravity aspects
Source: Sci Rep. 2023 Jul 28;13:12259. doi: 10.1038/s41598-023-39100-x (PMC10382528; doi:10.1038/s41598-023-39100-x)
Supplement: Supplementary file 1 — Supplementary Information. [file 41598_2023_39100_MOESM1_ESM.docx]

**Comparison Between the Geological Features of Venus and Earth Based on Gravity Aspects**

**Kurosh Karimi^1*^, Gunther Kletetschka^1,2^, Verena Meier^1^**

^1^ Institute of Hydrogeology, Engineering Geology and Applied Geophysics, Faculty of Science, Charles University, Prague 12843, Czech Republic.

^2^ Geophysical Institute, University of Alaska - Fairbanks, AK 99709 Fairbanks 903 N Koyukuk Drive, AK, USA

***** Corresponding author: Kurosh Karimi ([karimik@natur.cuni.cz](mailto:karimik@natur.cuni.cz))

**Appendix**

The gravity gradient tensor (GGT) or Marussi tensor is a multichannel dataset represented as a symmetric matrix with five independent components:

$\boldsymbol{\Gamma}= \nabla(\boldsymbol{\nabla}T)=\left[ \begin{matrix} \Gamma_{ii} & \Gamma_{ij} & \Gamma_{ik} \\ \Gamma_{ji} & \Gamma_{jj} & \Gamma_{jk} \\ \Gamma_{ki} & \Gamma_{kj} & \Gamma_{kk} \end{matrix} \right]$ (1)

where T is the disturbing potential, subscripts *ij* are two orthogonal components of the coordinate system (e.g., Cartesian), where each of “i” and “j” are x, y, z. The *ith* component of the gravity vector is $g_{i}=\frac{\partial T}{\partial i}$ and the second derivative components of the gravity potential are $\Gamma_{ij}=\frac{\partial}{\partial i}\left( g_{j} \right)= \frac{\partial}{\partial i}\left( \frac{\partial T}{\partial j} \right)= \frac{\partial^{2}T}{\partial i \partial j}=\Gamma_{ji}$.

There are five independent components in equation (1) for two reasons: (1) In a free source condition, i.e., the measurement is done out of the gravitational source, the Laplace equation holds, i.e., $\nabla^{2} T=0and\Gamma_{kk}=-(\Gamma_{ii}+\Gamma_{jj})$, and (2) $\Gamma$ is symmetric ($\Gamma_{ik}=\Gamma_{ki}, \Gamma_{ij}=\Gamma_{ji}, and \Gamma_{jk}=\Gamma_{kj}$). In this research, gravity disturbance and $\Gamma$ components are calculated from disturbing static gravitational potential in terms of spherical harmonics and are derived from Graflab software^1^. The disturbing potential is^2^:

$T\left( r,\varphi,\lambda\right)=\frac{GM}{r}\sum_{l=2}^{\infty} \sum_{m=0}^{l} ( {\frac{R_{p}}{r})}^{l}\left\{ C_{lm}^{'}cos m\lambda+S_{lm}sin m\lambda\right\} P_{lm}(sin \varphi)$ (2)

where G and M are the universal gravity constant and mass of the planet, respectively. R_p_ is the radius of the planet at the equator, r is the radial distance of a point outside the planet where T (disturbing potential) is calculated, $P_{lm}(sin \varphi)$ is a Legendre polynomial function, *l* and *m* are the degree and order of the harmonic expansion, respectively, and $\lambda\mathrm{and} \varphi$ are the geocentric longitude and latitude. $C_{lm}^{'}$and $S_{lm}$ are normalized Stokes parameters (harmonic geopotential coefficients), where $C_{lm}^{'}=C_{lm}-C_{lm}^{el}$, where $C_{lm}^{el}$ is the coefficient of the reference ellipsoid.

The spherical approximation of the gravity disturbance is^2^:

$\delta g=-\frac{\partial T}{\partial r}$ (3)

**Tensor components** **in Cartesian coordinate system**

In a Cartesian coordinate system, several of the GGT components relate to a crustal material-specific density distribution, and have the following properties:

$\Gamma_{zz}$: This component is the first vertical derivative of the vertical gradient of the disturbing potential ($\frac{\partial g_{z}}{\partial z}$) ($\Gamma_{zz}$is, in essence, a high pass filter with a strengthening property of the signals from shallow structures). $\Gamma_{zz}$ is the strongest signal to noise among all GGT components^3^{Citation}.

$\Gamma_{xz}, \Gamma_{yz}$: The horizontal derivatives of the vertical gradient of the disturbing potential in $\hat{x}$ and $\hat{y}$ directions ($\frac{\partial g_{z}}{\partial x}, \frac{\partial g_{z}}{\partial y} ).$ $\Gamma_{xz}, \Gamma_{yz}$ delineate the edges of the anomalous density structures or contact areas as$THG (\mathbf{T}\mathrm{otal}\mathbf{H}\mathrm{orizontal}\mathbf{G}radient)=\sqrt{{\Gamma_{xz}}^{2}+{\Gamma_{yz}}^{2}}$, which is invariant about the $\hat{\boldsymbol{z}}$ axis. When the contact dip between contrasting density volumes is vertical, THG indicates a clear edge structure, while in the case of gentle slopes, the magnitude of this parameter decreases towards the noise level^3^.

$( \Gamma_{xx}, \Gamma_{yy})$: Two horizontal derivatives ($\frac{\partial g_{x}}{\partial x}, \frac{\partial g_{y}}{\partial y} )$ of the horizontal gradients of the disturbing potential. Through Laplace equation, $\Gamma_{xx}+\Gamma_{yy}=-\Gamma_{zz}$.

${(\Gamma}_{xy}\mathrm{or}\Gamma_{yx})$ is a horizontal derivative of the horizontal component. The derivative and the component are perpendicular. $\Gamma_{xy}$can pinpoint the corners of the underground volumes of contrasting density.

**Invariants**

The tensor $\boldsymbol{\Gamma}$ has three invariants *I_0_, I_1_* and *I_2_*, meaning that under any coordinate rotation, their values do not change$:$

$I_{0}=Trace\left( \Gamma\right)=\sum_{i=1}^{3} \Gamma_{ii}=0$(4)

$I_{1}=\frac{1}{2}\left( (Trace {\left( \Gamma\right))}^{2}-Trace(\Gamma^{2}) \right)=\Gamma_{ii}\Gamma_{jj}+\Gamma_{ii}\Gamma_{kk}+\Gamma_{jj}\Gamma_{kk}-{\Gamma_{ij}}^{2}-{\Gamma_{jk}}^{2}-{\Gamma_{ik}}^{2}$ (5)

$I_{2}=det\left( \Gamma\right)=\Gamma_{ii}\left( \Gamma_{jj}\Gamma_{kk}- \Gamma_{jk}\Gamma_{kj} \right)+\Gamma_{ij}\left( \Gamma_{jk}\Gamma_{ki} - \Gamma_{ji}\Gamma_{kk} \right)+\Gamma_{ik}\left( \Gamma_{ji}\Gamma_{kj} - \Gamma_{jj}\Gamma_{ki} \right)$ (6)

Since $\boldsymbol{\Gamma}$ is a symmetric matrix, its eigenvectors and eigenvalues should be perpendicular and real, respectively. From this condition, it follows that^4^:

$0\leq I=-\frac{{{(I}_{2}/2)}^{2}}{{{(I}_{1}/2)}^{3}}\leq1$ (7)

This implies that I_1_ < 0 in any case.

*I_1_* and *I_2_* are two high pass filters amplifying the sources near the measurement point (surface) with units of s^-4^ and s^-6^, respectively. For example, for a point source ^4^ $I_{1}=-3\frac{{(Gm)}^{2}}{r^{6}}$ and $I_{2}=-2\frac{{(Gm)}^{3}}{r^{9}}$. Compared with $T_{zz}=\frac{Gm}{r^{2}}$, the strength of the filters in passing the high frequency signals are as follows:

*I_2_ > I_1_ >* $\Gamma$_zz_

This means that, increasing the distance, the *I_2_* weakens the deep anomalous sources faster than *I_1_*, and *I_1_* faster than $\Gamma$_zz_. The *I_1_* quantity is different from the other two in that this parameter cannot distinguish the negative sources from the positive ones. In other words, both positive and negative signals are boosted without showing their signs (*I_1_* is always negative). So, this feature could be regarded as a disadvantage. On the other hand, $\Gamma$_zz_ and *I_2_* maintain the signs of the anomalies (Figures 1 and 3 in the main text). These high pass filters should be treated with caution because they are more susceptible to noise as the power of “r” rises.

*I* can be called a “dimensionality indicator” ^5^ whose “zero” value shows a pure 2D distributed density, and value approaching “one” signifies a density distributed in 3D. A pure 2D body is a body in which one horizontal dimension goes to physical infinity and becomes much larger than the other horizontal dimension. In a pure 3D mass, the two horizontal dimensions of the body are exactly the same size; the vertical dimension could be smaller or larger. It should be noted, however, that although a causative body is 3-D, the determinant of GGT (*I_2_)* and *I* might be zero at some points of the measurement plane. Therefore, the zero value of *I* for the 2-dimensional state is a necessary condition but not sufficient. In other words, a 2-D mass has a zero value of *I*, but a zero value of *I* does not necessarily mean that the mass is 2-dimensional. In contrast, *I=*1 always signifies 3-dimensionality.

There is no distinct criterion for separation between 2- and 3-dimensionality. Some consider *I=*0.3 ^6^ and others consider *I=*0.5 ^5^ as a threshold for differentiating 2D from 3D bodies. The closer *I* is to unity, the closer the mass is to a pure 3-dimensional body like a sphere. A closer amount to zero could represent 2-dimensionality for cases when the determinant of GGT is zero. Take, for example, a long horizontal cylinder along the x axis (2D) in a Cartesian coordinate system. In such a case, the first row and column of $\boldsymbol{\Gamma}$ are zero, and I_2_ = I = 0.

$$\boldsymbol{\Gamma}=\left[ \begin{matrix} 0 & 0 & 0 \\ 0 & \boldsymbol{\Gamma}_{yy} & \boldsymbol{\Gamma}_{yz} \\ 0 & \boldsymbol{\Gamma}_{yz} & \boldsymbol{\Gamma}_{zz} \end{matrix} \right]$$

Note that 3- or 2-dimensionality also depends on the measurement point distance from the underground causative body as well as the grid data points. For instance, an anomalous structure such as the oceanic-continental plate boundary might seem 2-dimensional from a far measurement point (for example, at h=50 km from the reference ellipsoid), while it appears 3-D if we conduct airborne gravimetry at h=2 km with a grid network of $100 m \times100 m$.

**Strike Alignment (strike direction)**

Strike Alignment (SA) is a direction along which the gravitational response of a geological construct is constant. The strike direction could be parallel to the weakness in the configurational structure of rocks and masses, such as faults, and schistosity directions, or parallel to fold axis.

Consider the long horizontal cylinder in the previous section. When its alignment does not coincide with the ***i*** axis, the coordinate system should be rotated around the ***j*** axis in such an angle that in a least square sense, the rotated axis (***i’***) fits the strike direction of the body. This rotation angle yields the strike direction of the body ^4^. In the rotated coordinate system (**i’, j’, k’**):

$$\boldsymbol{\Gamma'}=\left[ \begin{matrix} {\Gamma'}_{ii} & {\Gamma'}_{ij} & {\Gamma'}_{ik} \\ {\Gamma'}_{ij} & {\Gamma'}_{jj} & {\Gamma'}_{jk} \\ {\Gamma'}_{ik} & {\Gamma'}_{jk} & {\Gamma'}_{kk} \end{matrix} \right]$$

To find the strike direction, $\theta_{s},$the objective function, $Q={{\Gamma^{'}}_{ii}}^{2}+{{\Gamma^{'}}_{ij}}^{2}+{{\Gamma'}_{ik}}^{2}$, should be minimal with respect to $\theta_{s}$ (modified from Pedersen and Rasmussen, 1990)^4^:

$$\frac{\partial Q}{\partial\theta_{s}}=\frac{\partial}{\partial\theta_{s}}[{{\Gamma^{'}}_{ii}}^{2}+{{\Gamma^{'}}_{ij}}^{2}+{{\Gamma'}_{ik}}^{2}]=0$$

$\theta_{s}=\frac{1}{2}\left\{ {tan}^{-1}(2 \frac{\Gamma_{ij}\left( \Gamma_{ii}+\Gamma_{jj} \right)+ \Gamma_{ik}\Gamma_{jk}}{{\Gamma_{ii}}^{2}-{\Gamma_{jj}}^{2}+{\Gamma_{ik}}^{2}-{\Gamma_{jk}}^{2}}) \right\}=\frac{1}{2}\left\{ {tan}^{-1}(2 \frac{{-\Gamma}_{ij}\Gamma_{kk}+ \Gamma_{ik}\Gamma_{jk}}{{\Gamma_{ik}}^{2}-{\Gamma_{jk}}^{2}+\Gamma_{kk}(\left( \Gamma_{ii}-\Gamma_{jj} \right))}) \right\}$ (8)

Note that $\theta_{s}$ could be computed within a multiple of $\frac{\pi}{2}$, i.e., (8) gives an extreme value for $Q$. Thus, $Q\left( \theta_{s} \right) and Q(\theta_{s}+\frac{\pi}{2}$) at each data point should be calculated, and the minimum value gives the true strike direction, provided that *I* is small. The strike direction could also be derived from the direction of the eigenvectors corresponding to the minimum eigenvalues of the GGT tensor^5^. It is worth mentioning that the intended coordinate system of Pederesen and Rasmussen (1990) ^4^ differs from the coordinate system that is utilized in this work. Consequently, equation (8) should be modified with respect to the adopted reference frame. We present the modified form after illustration of the various coordinate systems that are applied in this study.

Let (**X, Y, Z)** be the geocentric rectangular coordinates.

(**x, y, z** (or **r**)) is the Local North Oriented coordinates system, in which **x** points to the north, **y** to the west, and **z** (or **r**) to up (the radially outward direction from the center of the earth, Figure S1)).

(**e, n, z** (or **r**)) is a coordinate system, where **e** is directed toward the east, n toward the north and **z** radially outward (Figure S1).


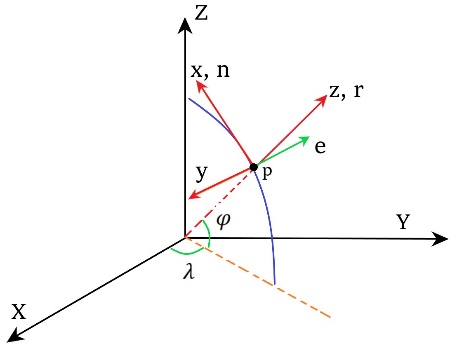


Figure S1: The relationship between different coordinate systems **(x,y,z)**, **(e,n,z)**, and **(X,Y,Z).**

The gravity vector in (**i, j, k**) coordinates is $\boldsymbol{g}=\left( \begin{aligned} g_{i} \\ g_{j} \\ g_{k} \end{aligned} \right)$, and its gradient is, $\nabla_{ijk}=\left( \begin{aligned} \frac{\partial}{\partial i} \\ \frac{\partial}{\partial j} \\ \frac{\partial}{\partial k} \end{aligned} \right).$ Here (**i, j, k**) could be any of (**e, n, z**), (**x, y, z**) and (**X, Y, Z**) coordinate systems.

$\Gamma_{ijk}= \nabla_{ijk} g^{T}=\left[ \begin{matrix} \Gamma_{ii} & \Gamma_{ij} & \Gamma_{ik} \\ \Gamma_{ji} & \Gamma_{jj} & \Gamma_{jk} \\ \Gamma_{ki} & \Gamma_{kj} & \Gamma_{kk} \end{matrix} \right]$ (9)

The relationship between the (enz) and (xyz) coordinate systems is:

$g_{enz}=\tau'g_{xyz}$ (10)

$\nabla_{enz}=\tau^{'} \nabla_{xyz}$ (11)

where $\tau'$ is a rotational matrix around $\hat{\boldsymbol{z}} (or \hat{\boldsymbol{r}} )$ as much as $\theta=\frac{\pi}{2} ADDIN ZOTERO\_ITEM CSL\_CITATION \{"citationID":"293XMVAq","properties":\{"formattedCitation":"\backslash\backslash super 4\backslash\backslash nosupersub\{\}","plainCitation":"4","noteIndex":0\},"citationItems":[\{"id":20,"uris":["http://zotero.org/users/11701102/items/C9DKGKFA"],"itemData":\{"id":20,"type":"article-journal","abstract":"The full gradient tensor is presently not measured routinely onboard airplanes or on land. This paper describes some improvements that can be made in strategies of data collection and in processing of potential field maps if such tensor measurements were available. We suggest that, in addition to producing for example standard total field anomaly maps, the invariants of the tensor be mapped. Strikes of magnetic or gravimetric structures may be determined from minimizing the power in the first row and column of the tensor. Invariants can be looked upon as nonlinear filters enhancing sources with big volumes. Their lateral resolution is superior to that of the field proper and, for a given resolution, the flight altitude and separation between flight lines can be increased compared with the standard mode of operation. In airborne surveys the distance between flight lines is normally much larger than the height above the ground. This may introduce severe aliasing effects in the direction perpendicular to the flight lines. By increasing the flight altitude, aliasing effects are reduced at the expense of lateral resolution which, however, may be improved by mapping the tensor invariants in addition to the magnetic field. The estimated gradient tensor from total field magnetic data over the Siljan impact region shows that the magnetic properties of the area are very nonuniform even from a height of 430 m above the topography. The nonlinear filters discriminate major anomalies into separate units.","container-title":"GEOPHYSICS","DOI":"10.1190/1.1442807","ISSN":"0016-8033, 1942-2156","issue":"12","journalAbbreviation":"GEOPHYSICS","language":"en","page":"1558-1566","source":"DOI.org (Crossref)","title":"The gradient tensor of potential field anomalies: Some implications on data collection and data processing of maps","title-short":"The gradient tensor of potential field anomalies","volume":"55","author":[\{"family":"Pedersen","given":"L. B."\},\{"family":"Rasmussen","given":"T. M."\}],"issued":\{"date-parts":[["1990",12]]\}\}\}],"schema":"https://github.com/citation-style-language/schema/raw/master/csl-citation.json"\}$^4^:

$$\tau^{'}=\left[ \begin{matrix} cos(\frac{\pi}{2}) & sin(\frac{\pi}{2}) & 0 \\ -sin(\frac{\pi}{2}) & cos(\frac{\pi}{2}) & 0 \\ 0 & 0 & 1 \end{matrix} \right]$$

Similarly, for (enz) and (XYZ):

$g_{XYZ}=\tau g_{enz}$ (12)

$\nabla_{XYZ}={\tau\nabla}_{enz}$ (13)

where $\tau$ is a transformation matrix between (**e, n, z**) and (**X, Y, Z**)^6^:

$$\tau=\left[ \begin{matrix} -sin(\lambda) & -cos(\frac{\pi}{2}-\varphi)cos(\lambda) & sin(\frac{\pi}{2}-\varphi)cos(\lambda) \\ cos(\lambda) & -cos(\frac{\pi}{2}-\varphi)\sin(\lambda) & \sin(\frac{\pi}{2}-\varphi)sin(\lambda) \\ 0 & sin(\frac{\pi}{2}-\varphi) & cos(\frac{\pi}{2}-\varphi) \end{matrix} \right]$$

From equations (10) and (13):

$\Gamma_{enz}= \tau^{'} \Gamma_{xyz} {\tau'}^{T}$ (14)

$\Gamma_{XYZ}=\tau\Gamma_{enz} \tau^{T}$ (15)


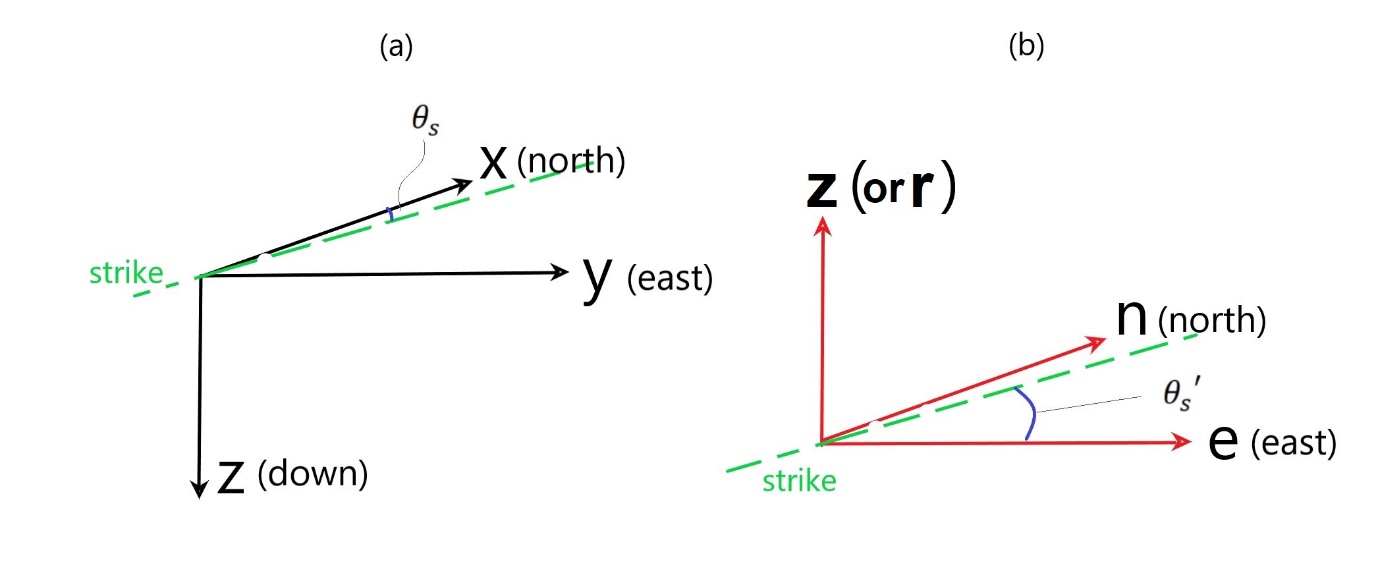


Figure S2: Relationships between the coordinate systems in the calculation of strike angles

In Pedersen and Rasmussen (1990)^4^, the coordinate system is right-handed (figure S2a), with **x** pointing to the north, **y** pointing to the east, and **z** pointing downward, and the strike angle is defined as (equation 17 in their paper):

$$tan(2\theta_{s})=2 \frac{\Gamma_{xy}\left( \Gamma_{xx}+\Gamma_{yy} \right)+ \Gamma_{xz}\Gamma_{yz}}{{\Gamma_{xx}}^{2}-{\Gamma_{yy}}^{2}+{\Gamma_{xz}}^{2}-{\Gamma_{yz}}^{2}}$$

where $\theta_{s}$ is the strike direction of a 2D structure with respect to the first component of the right-handed coordinate system, **x**. Their **(x,y,z)** coordinates is different from our **(x,y,z)** coordinates introduced in **Figure S1.**

The Marussi tensor components in this work are calculated in LNOF^1^. The strike direction could also be computed in any other right-handed coordinate system that is gained from the rotation of LNOF. We worked with (e, n, z), instead of LNOF (see Figure S2b).

If the disturbing gravity vector in (**x,y,z**) coordinates is $\boldsymbol{g}=\left( \begin{aligned} g_{x} \\ g_{y} \\ g_{z} \end{aligned} \right)$ and in (**e, n, z**) is $\mathbf{g}=\left( \begin{aligned} g_{e} \\ g_{n} \\ g_{z} \end{aligned} \right)$, we have:

$$g_{enz}=\tau g_{xyz}$$

$$\nabla_{enz}=\tau\nabla_{xyz}$$

$\boldsymbol{\Gamma}_{enz}= \tau\Gamma_{xyz} \tau^{T}$(16)

Where $\tau=\left[ \begin{matrix} 0 & 1 & 0 \\ 1 & 0 & 0 \\ 0 & 0 & -1 \end{matrix} \right]$, and

$$\Gamma_{ijk}= \nabla_{ijk} g^{T}=\left[ \begin{matrix} \Gamma_{ii} & \Gamma_{ij} & \Gamma_{ik} \\ \Gamma_{ji} & \Gamma_{jj} & \Gamma_{jk} \\ \Gamma_{ki} & \Gamma_{kj} & \Gamma_{kk} \end{matrix} \right] , \left( i,j,k \right)could be \left( e,n,z \right)or (x,y,z)$$

From (16), $\boldsymbol{\Gamma}_{enz}$ in terms of $\boldsymbol{\Gamma}_{\boldsymbol{xyz}}$ is:

$$\boldsymbol{\Gamma}_{enz}= \left[ \begin{matrix} \Gamma_{ee}=\Gamma_{yy} & \Gamma_{en}=\Gamma_{xy} & \Gamma_{ez}={-\Gamma}_{yz} \\ {\Gamma_{en}=\Gamma}_{xy} & \Gamma_{nn}=\Gamma_{xx} & \Gamma_{nz}={-\Gamma}_{xz} \\ \Gamma_{ez}={-\Gamma}_{yz} & \Gamma_{nz}={-\Gamma}_{xz} & \Gamma_{zz}=\Gamma_{zz} \end{matrix} \right]$$

Now, the angle that the strike creates with the east axis (${\theta_{s}}^{'}$) is obtained from (Figure S2b):

$\tan\left( 2{\theta_{s}}^{'} \right)=2 \frac{\Gamma_{en}\left( \Gamma_{ee}+\Gamma_{nn} \right)+ \Gamma_{ez}\Gamma_{nz}}{{\Gamma_{ee}}^{2}-{\Gamma_{nn}}^{2}+{\Gamma_{ez}}^{2}-{\Gamma_{nz}}^{2}}=2 \frac{\Gamma_{xy}\left( \Gamma_{yy}+\Gamma_{xx} \right)+(\left( -\Gamma_{yz} \right)\left( -\Gamma_{xz} \right))}{{\Gamma_{yy}}^{2}-{\Gamma_{xx}}^{2}+{\Gamma_{yz}}^{2}-{\Gamma_{xz}}^{2}}=-tan(2\theta_{s})$ (17)

$\theta_{s}$is the angle that the strike constructs with the **x** axis (Figure S2a)

From (16), it is concluded that ${\theta_{s}}^{'}={-\theta}_{s}+n\frac{\pi}{2}$

It should be noted here that Pedersen and Rasmussen ^4^ worked with $\theta_{s}$, but we work with ${\theta_{s}}^{'}$, which is complementary to $\theta_{s}$.

**Comb Factor**

CF^7^ is a tool to show how aligned the SA vectors are (Figure 2 in the main file). In fact, the direction of *θ* angle at each data point is sketched by a horizontal unit vector. A sliding square window is devised to include 9 adjacent data points. Arithmetic-mean of the scalar products between a vector in the center of the window with its 8 neighboring vectors is calculated and the result is attributed to that central point. Then the window is slid to encompass the next 9-member array. If the cosine of the angle of two neighboring vectors approaches 1 (~0-degree angle between the unit vectors), alignment is the maximum. In opposite, when the scalar product of these vectors is close to zero, the neighboring vectors are misaligned to maximum degree, close to perpendicular to each other. As a threshold, CF > 0.98 is selected for indicating a good alignment characteristic, and CF < 0.98 is adopted to showing no alignment (Figure 2 in the main file).

**Studied areas on the planets**

The considered areas in this study in terms of gravity aspects are depicted on topography maps of the Earth and Venus in Figures 3a and 3b, respectively.


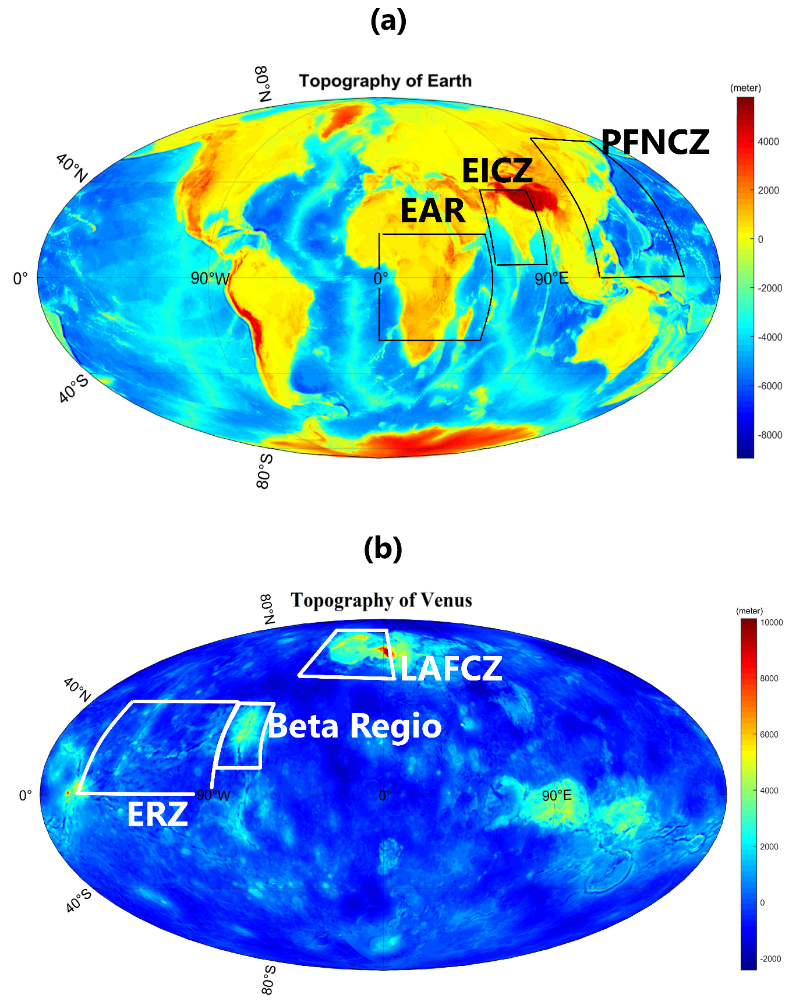


Figure S3: Topography of (a) Earth; (b) Venus. The examined areas in terms of gravity aspects in the main file- East African Rift (EAR), Pacific and Filipino-North American contact zone (PFN CZ), Eurasia and Indian contact zone (EICZ), equatorial rifting zone (ERZ), Beta Regio and Lakshmi planum- Akna Montes-Freyja Montes contact zone (LAFCZ)- are marked.

**References**

1. Bucha, B. & Janák, J. A MATLAB-based graphical user interface program for computing functionals of the geopotential up to ultra-high degrees and orders. *Computers & Geosciences* **56**, 186–196 (2013).

2. Heiskanen, W. A. & Moritz, H. Physical geodesy. *Bull. Geodesique* **86**, 491–492 (1967).

3. Zengerer, M. An Overview of Tensors, Gradient and Invariant Products in Imaging and Qualitative Interpretation. *ASEG Extended Abstracts* **2018**, 1–8 (2018).

4. Pedersen, L. B. & Rasmussen, T. M. The gradient tensor of potential field anomalies: Some implications on data collection and data processing of maps. *GEOPHYSICS* **55**, 1558–1566 (1990).

5. Beiki, M. & Pedersen, L. B. Eigenvector analysis of gravity gradient tensor to locate geologic bodies. *GEOPHYSICS* **75**, I37–I49 (2010).

6. Tsunakawa, H., Takahashi, F., Shimizu, H., Shibuya, H. & Matsushima, M. Surface vector mapping of magnetic anomalies over the Moon using Kaguya and Lunar Prospector observations. *Journal of Geophysical Research: Planets* **120**, 1160–1185 (2015).

7. Kletetschka, G. *et al.* Distribution of water phase near the poles of the Moon from gravity aspects. *Sci Rep* **12**, 4501 (2022).
